# A quantitative comparison between the essential medicines for rheumatic diseases in children and young people in Africa and the WHO model list

**DOI:** 10.1186/s12969-024-00997-x

**Published:** 2024-07-04

**Authors:** Waheba Slamang, Christiaan Scott, Helen E. Foster

**Affiliations:** 1 University of Cape Town, Cape Town, South Africa; 2 Paediatric Rheumatology European Society Global Health Research Fellow 2022, Cape Town, South Africa; 3 Newcastle University, Newcastle Upon Tyne, UK; 4 University of Ottawa, Ottawa, Canada

**Keywords:** Essential medicines, Africa, WHO, Juvenile rheumatic diseases, Joint diseases, Children

## Abstract

**Background:**

The World Health Organisation Essential Medicines List (WHO EML) guides National Essential Medicines Lists and Standard Treatment Guidelines for clearly identified disease priorities especially in low- and middle-income countries. This study compares the degree to which the basket of medicines recommended for rheumatic diseases in children and young people in National Essential Medicines Lists of countries in the WHO Africa region, corresponds to the 2021 WHO EML and WHO EML for children, as a proxy of availability.

**Methods:**

An online search of the WHO medicines and health technology portal, the Health Ministry websites of the 54 African countries, PUBMED and Google Scholar, with search terms for ‘National Essential Medicines List’, AND/OR ‘standard treatment guidelines’ AND/OR ‘Lista Nacional de Medicamentos Essenciais’ AND/ OR ‘Liste Nationale de Medicaments Essentiels’ AND Africa AND/OR < Name of African country > was conducted. The number of medicines on the national lists were compared according to a predefined template of medicines; and the percentage similarity calculated. Descriptive statistics were derived using STATA.

**Results:**

Forty-seven countries in the WHO Africa region have developed a National Essential Medicines List. Eleven countries do not have any medicines listed for rheumatic diseases.

The majority of countries had less than or equal to 50% similarity with the WHO EML for rheumatic disease in children and young people, median 3 medicines (IQR 1— 4). The most common medicines on the national lists from Africa were methotrexate, sulfasalazine and azathioprine, with etanercept available in 6 countries. Seven countries had only one medicine, acetylsalicylic acid listed in the section ‘Juvenile Joint diseases’.

A multiple linear regression model for the predictors of the number of medicines on the national lists established that 20% of the variability was predicted by health expenditure per capita, socio-demographic index and the availability of rheumatology services (adult and/or paediatric) *p* = 0.006, with socio-demographic index (*p* = 0.035, 95% CI 0.64—16.16) and the availability of rheumatology services (*p* = 0.033, 95% CI 0.13 – 2.90) significant.

**Conclusion:**

Four countries (8.5%) in Africa have updated their National Essential Medicines Lists to reflect adequate care for children and young people with rheumatic diseases. Moving forward, efforts should focus on aligning available medicines with the WHO EML, and strengthening healthcare policy for rheumatology and pharmaceutical services, for affordable access to care and medicines.

**Supplementary Information:**

The online version contains supplementary material available at 10.1186/s12969-024-00997-x.

## Background

The Global Burden of Disease (GBD) study has consistently provided data in support of the increase in prevalence of musculoskeletal disorders in Africa [1]. Rheumatic diseases in children and young people (CYP) are a considerable source of musculoskeletal pain and disability, and are included in this burden; the chronic disabling effects often noted throughout the peak education, career defining and income earning years [2–4]. Poorer health outcomes are associated with low gross domestic product; and increased disease activity and damage are attributable to delayed access to healthcare, late diagnosis, and the use of prolonged corticosteroids in the absence of appropriate medicines [5]. Compounded by the necessary prioritisation of communicable diseases, lack of healthcare funding, poverty, and other social determinants, the burden of rheumatic diseases in CYP is often underestimated and overlooked; and access to the necessary medicines and care highly variable [6, 7].

The equitable provision of medicines is an essential component of the modified World Health Organisation (WHO) health systems strengthening building blocks [8, 9] and has been included in the Global Strategy for Musculoskeletal Health [10]. The World Health Organisation Essential Medicines List (WHO EML) supports these frameworks and improves health care for identified disease priorities, by streamlining access to medicines in safe and cost-effective ways. The evidence-based policy document informs national essential medicines lists (NEMLs) and standard treatment guidelines (STGs) [11, 12]. Since its inception in 1977, the WHO EML has expanded to include over 500 medicines organised alphabetically by indication and has evolved to provide a *core* and *complementary* model list of essential medicines. The *core* list presents the minimum medicines needed for priority conditions to enable a functional basic healthcare system. The *complementary* list presents medicines for priority diseases, for which specialised diagnosis and monitoring facilities and/or specialist medical care is needed; therapeutic alternatives are indicated on the lists by a square box.

The development of the WHO EML for children (WHO EMLc) in 2007, acknowledged the unique health needs of the paediatric population, and is defined for children up to and including 12 years of age. Children older than 12 years are intended to access medicines on the WHO EML. The lists are revised every 2 years, to ensure the provision of updated, effective medicines. By 2017, 137 WHO member states had developed NEMLs in efforts towards establishing Universal Health Coverage [13]. The WHO model lists are thus useful tools to assess access and availability of medicines for specific diseases across countries [14].

The Global Paediatric Musculoskeletal Task Force (TF), a virtual community of individuals involved in paediatric rheumatology care, was established in 2017 and aims to improve access to ‘right care’ to gain better outcomes for CYP with musculoskeletal disorders [15, 16]. The TF identified the lack of access to medicines to treat rheumatic diseases in CYP as a specific unmet need undermining care in low- and middle-income countries (LMIC) [16–18] and noted that the 2019 WHO EML and EMLc were not aligned with current standard care for juvenile rheumatic disorders. Methotrexate and hydroxychloroquine were listed in Sect. 29.2 disease-modifying anti-rheumatic drugs (DMARDs) and only acetylsalicylic acid was noted in Sect. 29.3 ‘Juvenile joint diseases’. Tumour necrosis factor inhibitors (TNFi) were listed separately in Sect. 8.1 ‘Immunomodulators’; and were not clearly identified for use in juvenile joint diseases [19].

Following engagement with the global community involved in paediatric rheumatology care and subsequent published e-surveys to gauge opinion on which medicines should be included in the WHO EML, the 2020 and 2022 applications by the TF to update the WHO EML for rheumatic diseases in CYP, has resulted in important changes [18–20].

The 2021 WHO EML and EMLc demonstrates improved signposting to the medicines available for the treatment of ‘Juvenile joint diseases’ and the recently released 2023 versions, clearly lists the medicines on the complementary list in this section (Table 1). Triamcinolone hexacetonide, with triamcinolone acetonide as an alternative for locoregional intra-articular joint injections are the only new additions in 2023. These changes were necessary to reflect modern management of juvenile joint diseases for more effective advocacy. The adult EML (applicable to children older than 12 years) includes additional DMARDs and the TNFi certolizumab pegol and golimumab as alternatives. Glucocorticoids and non-steroidal anti-inflammatory drugs are not specifically listed for the treatment of rheumatic diseases in children but are present under the sections for ‘Anti-allergics and medicines used in anaphylaxis’, ‘Hormones and antihormones’ and ‘medicines for pain and palliative care’. Notably absent are Interleukin-1 and Interleukin-6 inhibitors which have been shown to improve outcomes in the pathophysiologically distinct subtype of systemic onset juvenile idiopathic arthritis (JIA) [21–23].

**Table 1:** WHO EML/ EMLc 2023 Medicines for rheumatic diseases in children and young people

WHO EMLc	Dosage form
Section 29 Medicines for Diseases of Joints	
Section 29.2 Disease-modifying anti-rheumatic drugs	
Methotrexate	Oral > Solid > tablet: 2.5 mg (as sodium salt)
Hydroxychloroquine	Oral > Solid: 200 mg (as sulphate)
Azathioprine	Oral > Solid > tablet: 50 mg (scored); 25 mg
Section 29.3 Medicines for Juvenile Joint Diseases	
Acetylsalicylic acid	Oral > Solid: 100 to 500 mg Local > Rectal > Suppository: 50 to 150 mg
Biologic DMARDs	
Adalimumab	Parenteral > General injections > SC: 40 mg per 0.8 mL; 40 mg per 0.4 mL; 10 mg per 0.2 mL; 20 mg per 0.4 mL
*Therapeutic Alternatives*	
Etanercept	Parenteral > General injections > SC: 25 mg per 0.5 mL; 50 mg per 1.0 mL
Infliximab	Parenteral > General injections > IV: 100 mg vial 10 mg per 1.0 mL
Locoregional Joint Injection	
Triamcinolone hexacetonide	*Injection: 20 mg/mL in vial*
*Alternative*: Triamcinolone acetonide	
WHO EML	Dosage Form
Section 29 Medicines for diseases of joints	
Section 29.2 Disease-modifying anti-rheumatic drugs	
Azathioprine	Oral > Solid > tablet: 50 mg (scored); 25 mg
^c^Chloroquine	Oral > Solid: 100 mg tablet (as phosphate or sulfate); 150 mg tablet (as phosphate or sulfate)
Hydroxychloroquine	Oral > Solid: 200 mg (as sulfate)
Methotrexate	Oral > Solid > tablet: 2.5 mg (as sodium salt)
Penicillamine	Oral > Solid: 250 mg
Sulfasalazine	Oral > Solid: 500 mg
Section 8.1 Immunomodulators for non-malignant diseases	
Adalimumab	Parenteral > General injections > SC: 40 mg per 0.8 mL; 40 mg per 0.4 mL; 10 mg per 0.2 mL; 20 mg per 0.4 mL
*Therapeutic Alternatives*	
Certolizumab pegol	Parenteral > General injections > SC: 200 mg per 1.0 mL
Etanercept	Parenteral > General injections > SC: 25 mg per 0.5 mL; 50 mg per 1.0 mL
Golimumab	Parenteral > General injections > SC: 50 mg per 0.5 mL; 45 mg/0.5 ml > IV: 50 mg/4 ml
Infliximab	Parenteral > General injections > IV: 100 mg vial 10 mg per 1.0 mL
WHO EML Sect. 29.3 Juvenile Joint Diseases	
Acetyl salicylic acid	Oral > Solid: 100 to 500 mg Local > Rectal > Suppository: 50 to 150 mg
Methotrexate	Oral > Solid > tablet: 2.5 mg (as sodium salt)
Adalimumab	Parenteral > General injections > SC: 40 mg per 0.8 mL; 40 mg per 0.4 mL; 10 mg per 0.2 mL; 20 mg per 0.4 mL
*Therapeutic Alternatives*	
Certolizumab pegol	Parenteral > General injections > SC: 200 mg per 1.0 mL
Etanercept	Parenteral > General injections > SC: 25 mg per 0.5 mL; 50 mg per 1.0 mL
Golimumab	Parenteral > General injections > SC: 50 mg per 0.5 mL; 45 mg/0.5 ml > IV: 50 mg/4 ml
Infliximab	Parenteral > General injections > IV: 100 mg vial 10 mg per 1.0 mL
Locoregional Joint Injection	
Triamcinolone hexacetonide	Injection: 20 mg/mL in vial
*Alternative*: Triamcinolone acetonide	

*Abbreviations*: *mg* milligrams, *mL* millilitres, *SC* subcutaneous, *IV* intravenous

In Africa 40% of the population are children under 15 years old [24]. Considering the increasing burden of musculoskeletal disorders (including rheumatic diseases) in Africa, and previously reported poorer outcomes in these regions as a result of poor access and availability of appropriate treatment for rheumatic diseases in CYP [1, 5], the provision of the necessary medicines and care are essential. The WHO EML and EMLc are invaluable policy documents to achieve this.

This article compares the NEMLs and STGs available for countries in the WHO Africa region, with the 2021 WHO EML and EMLc, and focuses on medicines listed for the treatment of rheumatic diseases in CYP as a proxy for availability. Novel strategies to provide holistic care and medicines in the quest for Universal Health Coverage for CYP with rheumatic diseases are further considered.

## Methods

A systematic, targeted online search of the 54 countries constituting the WHO Africa region was conducted in the WHO medicines and health products portal country profiles, (including the WHO Institutional Repository for Information Sharing and Global Index Medicus), for NEMLs and STGs. The publicly accessible online Ministry of Health website for each country was searched for the latest available version of these documents. A further search in PUBMED and Google scholar using the search terms ‘National Essential Medicines List’, AND/OR ‘standard treatment guidelines’ AND/OR ‘Lista Nacional de Medicamentos Essenciais’ AND/ OR ‘Liste Nationale de Medicaments Essentiels’ AND Africa AND/OR < Name of African country > was conducted, to maximise the number of valid documents obtained.

The most recently released documents obtainable online were used for this study.

### Inclusions

National Essential Medicines Lists were defined as: The list of medicines determined by the National Essential Medicines List Committee (NEMLC) appointed by the Minister of Health and maintained by the Essential Drug Program (EDP) of a country, deemed to satisfy the priority health care needs of the population [25]. Standard treatment guidelines were defined as: The implementation mechanism of the EML which provides guidance to health care professionals on the use of medicines which appear on the EML and contains background information on the disorders listed, treatment regimens, as well as other relevant information [25].

The medicines available on each of the NEMLs and noted in STGs for rheumatic diseases in CYP were compared to the basket of medicines available on the 2021 WHO EML and/or WHO EMLc (the 2021 version was the most recently available at the time of data extraction). To standardise comparisons and minimise the risk of missing data, the medicines were organised by section into a template for data extraction. (Supplementary Material). Comparisons were made between:

The National Essential Medicines Lists for children (NEMLc) and the 2021 WHO EML for children (WHO EMLc); the NEMLs for adults (where this was differentiated from the list for children) and the 2021 WHO EML, which is defined for adults and children > 12 years.

Where a country had developed a composite NEML for both adults and children, this was compared to the 2021 WHO EML and the section ‘Juvenile Joint Diseases’ additionally analysed. If both an NEML and NEMLc were developed by a country then both were included; i.e. medicines listed for rheumatic indications on all lists were included as these may all potentially be used to treat children and young people. Additional medicines, those not appearing on the WHO EML were also recorded.

The NEMLs were hand searched for the defined medicines and extracted data were organised using the template decided a priori. (Supplementary Material). The % similarity with the WHO EML was calculated as the:

$$(no.\;of\;medicines\;on\;NEML\div no.\;of\;medicines\;on\;WHO\;EML\;template\;list)\;\times\;100$$

Descriptive statistics were employed using STATA. A linear regression model was created to test the predictors health expenditure per capita, socio-demographic index, and the availability of paediatric and/or adult rheumatology services, on the number of medicines on the NEML. It is noteworthy that Gross Domestic Product has previously been shown *not* to predict the number of medicines on NEMLs [13] and was not included as a potential predictor in our analysis.

### Exclusions

Drug formularies that did not meet the definition of a NEML, and the WHO EML Sect. 29.1 – ‘Medicines for the treatment of gout’, as this is rarely encountered in children, were excluded. Several medicines used in oncology may also have a rheumatological indication e.g. methotrexate and rituximab. These medicines may be listed under immunomodulators and were excluded if not specifically noted for rheumatic diseases in the NEMLs.

## Results

### Overview (Table 2)

**Table 2:** Summary of country characteristics and comparison to WHO model list of essential medicines

*Countries with an NEML N* = *47*	*SDI 2019 GBD Study* [1]	*2019 Health Expenditure per capita (Int-$)*[26]	*2017 GDP per capita (Int-$)* [26]	*Countries with Adult and/or Paediatric Rheum service* [27]* N* = *28*	*Countries with established Paediatric rheum service* [27]* N* = *12*	*Countries with STGs N* = *21*	*NEML Year*	*No. of meds on NEML*	*Countries with NEML Section Juvenile Joint Diseases N* = *8*	*No. of meds on NEML Section Juvenile Joint Diseases*	*Countries with NEMLc N* = *7*	*No. of meds on NEMLc*
Algeria	0,63	751,00	13210,00	√	√	X	2016	4	X	-	X	-
Angola	0,42	178,00	6974,00	√	X	X	2021	6	√	1	X	-
Benin	0,32	82,00	4056,00	√	√	X	2018	4	X	-	√	2
Botswana	0,61	1122,00	18323,00	√	X	X	2016	3	X	-	X	-
Burkina Faso	0,24	122,00	25546,00	√	X	X	2020	2	X	-	√	0
Burundi	0,27	62,00	836,00	X	X	X	2012	0	X	-	X	-
Cameroon	0,46	136,00	4408,00	√	X	X	2017	0	X	-	X	-
Cape Verde	0,50	370,00	9083,00	X	X	X	2018	4	X	-	X	-
Central African Republic	0,26	78,00	967,00	√	X	X	2017	0	X	-	X	-
Chad	0,22	69,00	1668,00	X	X	X	2022	0	X	-	√	0
Congo	0,54	82,00	3791,00	X	X	X	2013	4	X	-	X	-
Côte d'Ivoire	0,38	180,00	6538,00	√	X	X	2020	0	X	-	X	-
Djibouti	0,43	104,00	5893,00	X	X	X	2007	0	X	-	X	-
Democratic Republic of Congo	0,34	41,00	1334,00	√	X	X	2020	2	X	-	√	0
Egypt	0,63	582,00	15091,00	√	√	X	2018	5	X	-	X	-
Eritrea	0,37	82,00	1629,00	X	X	X	2010	2	X	-	X	-
eSwatini	0,56	611,00	10782,00	X	X	√	2012	1	X	-	X	-
Ethiopia	0,30	75,00	2812,00	√	X	X	2020	5	√	1	X	-
Ghana	0,52	193,00	6498,00	√	√	√ ^i^	2019	10	X	-	X	-
Guinea	0,30	119,00	3187,00	X	X	X	2012	3	X	-	X	-
Kenya	0,48	208,00	5764,00	√	√	√ ^i^	2019	7	√	6	X	-
Lesotho	0,48	313,00	2695,00	X	X	√	2005	0	X	-	X	-
Liberia	0,34	126,00	1725,00	X	X	√	2017	6	√	1	X	-
Libya	0,71	409,00	23375,00	√	√	X	2019	8	√	1	X	-
Madagascar	0,37	65,00	1774,00	√	X	X	2008	3	X	-	X	-
Malawi	0,36	82,00	1732,00	X	X	√	2015	1	√	1	X	-
Mali	0,24	95,00	2517,00	√	√	X	2019	5	X	-	X	-
Mauritania	0,47	187,00	6424,00	√	X	√	2012	0	X	-	X	-
Morocco	0,52	425,00	9519,00	√	X	X	2014	4	X	-	X	-
Mozambique	0,28	105,00	1468,00	√	X	X	2017	4	X	-	X	-
Namibia	0,59	867,00	11205,00	X	X	√	2016	5	X	-	X	-
Nigeria	0,49	162,00	5869,00	√	√	√	2020	4	√	1	√	5
Rwanda	0,40	146,00	2792,00	X	√	√ ^i^	2015	1	X	-	√	2
Senegal	0,36	145,00	4209,00	√	√	X	2018	0	X	-	X	-
Seychelles	0,72	1469,00	35228,00	X	X	√	2010	2	X	-	X	-
Sierra Leone	0,32	157,00	1931,00	X	X	√	2019	0	X	-	X	-
Somalia	0,08	12,00	1364,00	X	X	√	2019	2	X	-	X	-
South Africa	0,66	1187,00	15905,00	√	√	√^i^	2023	4	X	-	√	5
South Sudan	0,34	63,00	1182,00	X	X	√	2018	3	X	-	X	-
Sudan	0,47	205,00	4216,00	√	√	√	2014	5	X	-	X	-
Tanzania	0,39	99,00	3097,00	√	X	√^i^	2021	4	√	1	X	-
The Gambia	0,37	89,00	2510,00	X	X	√	2001	0	X	-	X	-
Togo	0,39	125,00	2608,00	√	X	X	2012	4	X	-	X	-
Tunisia	0,65	789,00	12488,00	√	√	X	2016	9	X	-	X	-
Uganda	0,37	92,00	2694,00	√	X	X	2016	3	X	-	X	-
Zambia	0,47	193,00	3894,00	√	X	√	2020	1	X	-	X	-
Zimbabwe	0,45	208,00	2531,00	X	X	√ ^i^	2015	3	√	2	X	-

Table 2 presents a summary of the individual country characteristics, the availability of an NEML and/or NEMLc, STGs and notes the availability of rheumatology services as well as the number of medicines if present

*NEML* National Essential Medicines List, *NEMLc* National Essential Medicines List for children, *GDP* Gross Domestic Product, *GBD* Global Burden of Disease, *Int-$* International Dollars, *Rheum* Rheumatology, *SDI* Socio-demographic Index, *STG* Standard Treatment Guidelines, *√* available, *Meds* Medicines *X* not available, *i* paediatric rheumatic diseases noted in STGs

Forty-seven countries in the WHO Africa region had an NEML. The NEML for Gabon was not available online and was excluded. In South Africa, the primary care, paediatric- and adult-hospital level STGs linked to the NEML were included. Eleven countries had no medicines listed for rheumatic diseases. Eight countries had an NEML section for ‘Juvenile joint diseases’. Of the 7 countries with an NEMLc, 3 included a section for ‘Juvenile joint diseases’. Twenty-one countries (all in sub-Saharan Africa) had developed STGs linked to the NEML. Rwanda and South Africa had additionally formulated separate STGs for children. Overall, 6 countries included treatment for juvenile rheumatic diseases in the STGs.

#### Comparison of national EMLs and STG’s to WHO model lists

**Fig. 1:**
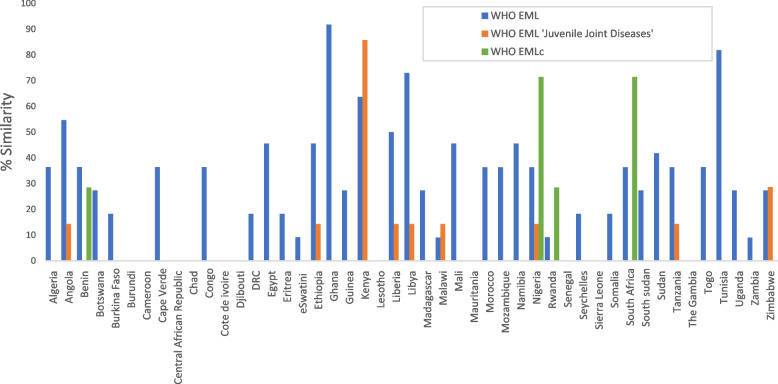
% Similarity between NEML and WHO Model Lists for rheumatic diseases. *The percentage* similarity between the NEML and the section Juvenile Joint diseases and the WHO EML; the NEMLc and the WHO EMLc, are represented for each country. 11 countries have no medicines for rheumatic diseases listed. 15% of countries have > 50% similarity with the WHO model lists. NEML: National Essential Medicines List; NEMLc: National Essential Medicines List for children, WHO EML: World Health Organisation Essential Medicines List; WHO EMLc: World Health Organisation Essential Medicines List for children

The majority (85%) of countries had less than or equal to 50% similarity with the WHO EML. The median no. of medicines recommended on the NEMLs were 3 (IQR 1–4), with listings for Ghana (91.7%) and Tunisia (83.3%) showing the highest similarity (Fig. 1). The medicines listed most commonly on the NEML were conventional synthetic DMARDs methotrexate, sulfasalazine and azathioprine. Least common were the TNFi, with etanercept recommended in six countries. (Supplementary Material).

Only one medicine, acetyl salicylic acid was listed in 7 of the 8 NEMLs with the section ‘Juvenile joint diseases’. Kenya had the highest similarity, listing 6 of the 7 medicines in this section of the NEML.

Ghana, Libya, and Kenya had the highest number of additional medicines for rheumatic diseases on their NEMLs, with ciclosporin, cyclophosphamide, and mycophenolate mofetil the most frequently added.

Acetylsalicylic acid and methotrexate were the most common medicines listed on the NEMLc. Nigeria and South Africa were the only two countries with tumour necrosis factor inhibitors (etanercept and adalimumab respectively) on their NEMLc, and who recommend additional medicines compared to the WHO EMLc.

**Fig. 2:**
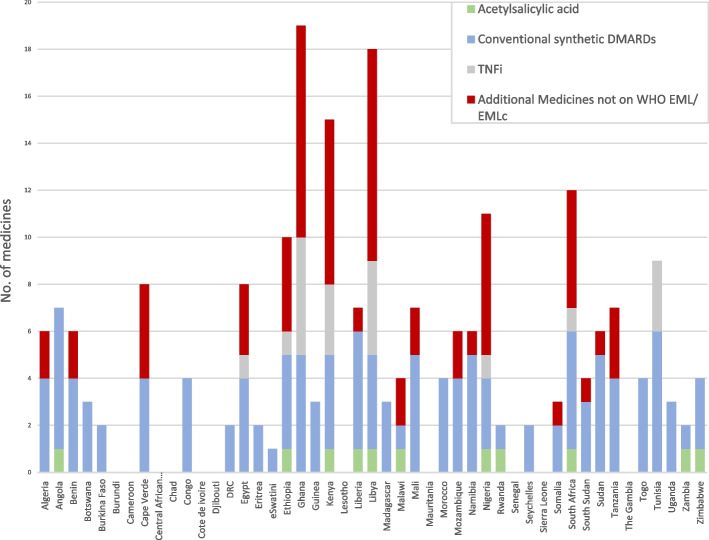
Summary of the essential medicines for rheumatic diseases in children and young people on NEMLs in African countries. The number of countries in which these medicines are listed are indicated in brackets. DMARDs: disease-modifying anti-rheumatic drugs; WHO EML: World Health Organisation Essential Medicines List; WHO EMLc: World Health Organisation Essential Medicines List for children. Conventional synthetic DMARDs may include methotrexate (33), and/or hydroxychloroquine (19), chloroquine (13), azathioprine (24), penicillamine (10), sulfasalazine (26); TNFi: tumour necrosis factor inhibitors may include adalimumab (4), and/or etanercept (6), infliximab (4), certolizumab pegol (2), golimumab (2). The additional medicines not on the WHO EML most commonly included ciclosporin (15), cyclophosphamide (10), mycophenolate mofetil (10), rituximab (5) leflunomide (5), tacrolimus (5), triamcinolone acetonide (3) abatacept (2) and tocilizumab (2). Anakinra was only listed in Libya, while belimumab and tofacitinib were only listed in Ghana

Figure 2 summarises the essential medicines available on NEMLs to treat rheumatic diseases in CYP in the WHO Africa region.

Multiple linear regression for the predictors of the number of medicines listed on the NEML were conducted for the country factors: health expenditure per capita, sociodemographic index and the availability of rheumatology services (paediatric and/or adult). The model established that 20% of the variance in the number of medicines listed may be predicted by the country factors as noted *p* = 0.006. The individual factors were examined further and indicated the significance of socio-demographic index (β-coefficient = 8.4 *p* = 0.035, 95% CI 0.64 – 16.2) and the availability of paediatric and/or adult rheumatology services (β-coefficient = 1.5 *p* = 0.033, 95% CI 0.13 – 2.90).

Similarly, a model for the predictors of TNFi on the NEMLs, resulted in *p* = 0.003, adj. R^2^ = 0.23, with socio-demographic index (β-coefficient = 5.14, *p* = 0.003, 95% CI 1.83 – 8.45) considered significant. The availability of rheumatology services was not significant in this model *p* = 0.10 (95% CI -0.99 – 1.08).

Health expenditure per capita was not significant in either of the models *p* = 0.45 (95% CI 0.005 – 0.002) and *p* = 0.08 (95% CI -0.003 – 0.0001) respectively.

## Discussion

The increasing reports and estimates of rheumatic diseases in CYP from African countries challenges the perception that these diseases are rare or non-existent in the WHO Africa region [6, 7, 27]. It necessitates an investment in healthcare for chronic inflammatory musculoskeletal disorders, and the provision of appropriate medicines to significantly improve quality of life and to reduce disability [28, 29].

As NEMLs inform the procurement and supply of medicines to meet priority healthcare needs in the public sector, the collation and comparison of NEMLs to the WHO EML for this study, highlights this aspect of care. It provides the data and impetus for stakeholders to advocate for updating their NEML to improve access and availability of the necessary medicines to treat CYP with rheumatic diseases, and simultaneously fuels discussions around financial risk protection for patients and their families in this setting.

Whilst work is ongoing to list medicines for systemic juvenile idiopathic arthritis i.e. tocilizumab and anakinra on the WHO EML, the latest versions of the model lists provide timely guidance for African countries to update their NEMLs to reflect standard care for rheumatic diseases in CYP. Only 4 countries have updated their NEMLs since 2021 and the majority of countries have a 50% or lower similarity with the 2021 WHO essential medicines model lists.

Conventional synthetic DMARDs (including the additional medicines not on the WHO EML i.e. mycophenolate mofetil, ciclosporin, and cyclophosphamide most commonly) appear to be more available and presumably due to their utility in adult rheumatic diseases; only 8 countries (South Africa, Ethiopia, Ghana, Nigeria, Kenya, Libya, Tunisia and Egypt,) listed biologic DMARDs, in keeping with more modern treatment approaches. Tumour necrosis factor inhibitors, as reflected in the WHO model lists, are noted for these 8 countries. The additional biologic DMARDs not listed on the WHO EML include tocilizumab in Nigeria, Libya and Kenya; rituximab in these 3 countries as well as in Ghana and South Africa; and anakinra only in Libya. Biologic DMARDs, while currently more costly than conventional synthetic DMARDs, have the potential to improve outcomes significantly [30, 31].

The provision of medicines is, however, dependent on adequate financing and robust pharmaceutical services [32], and must be adequately supported by health care policy. Notably, health care policy around priority diseases influences the development of NEMLs and STGs, and consequently the availability of medicines for patients managed by state health care services in particular. At the time of writing, there were no national healthcare policies in place for musculoskeletal healthcare in Africa, with only 6 countries in sub-Saharan Africa including the management of rheumatic diseases for CYP in STGs [33]. The expansion of rheumatology services across Africa [7, 27, 34, 35], supported by the clinician-led Paediatric society of the African League Against Rheumatism (PAFLAR) and the Global Paediatric Musculoskeletal Task Force, has provided evidence to address this challenge [5, 7, 15, 18, 20, 36]. However, to lever long term sustainable change, due consideration, coordination and funding at policymaker level is crucial. Furthermore adequate support for rheumatology services and the array of factors that influence access to appropriate care also need to be addressed; the Global Strategy for Musculoskeletal Health and the 2022 Australian ‘Inquiry into childhood rheumatic diseases’ describe strategies and adaptable exemplars for change at policymaker level, which may be implemented by the countries in Africa [10, 37].

However, the overall cost of treating rheumatic diseases in children, i.e. increased numbers of hospital visits, associated medical and non-medical costs including the high cost of biologic DMARDs, remains a major challenge [38–40]. Additionally, the main method of payment for about one third of healthcare services in the Africa region are financed non-sustainably and by ‘out-of-pocket’ expenditure [41]. These costs could be countered to some extent by improved clinical outcomes, the reduced costs of surgery, as well as the unmeasured longer term effects of chronic illness on mental health, and the impact on future productivity in society [42]. We therefore considered country specific factors including health care financing, which may affect the number of medicines on NEMLs and by proxy, their availability i.e. health expenditure per capita, socio-demographic index (a composite calculation of health expenditure per capita, average years of schooling and total fertility rate for females under 25 years), and the availability of rheumatology services, as predictors. As Gross Domestic Product has previously been shown not to predict the number of medicines on NEMLs [13, 14], this was not re-evaluated.

In our analysis, health expenditure per capita per se, did not predict the number of medicines on the NEMLs for rheumatic diseases in CYP. However, the availability of rheumatology services and socio-demographic index (SDI), were significant predictors of the number of medicines available, and the presence of tumour necrosis factor inhibitors on NEMLs.

The 2019 Global Burden of Disease study reported the median SDI for countries in the WHO Africa region as 0.41 (IQR 0.34–0.52) and ranged from 0.081 (Somalia) to 0.724 (Seychelles), with 56% of countries in the low SDI range. This measure is indicative of poorer health outcomes in the region, which may be further affected by a complex array of challenges [1]. While the 8 countries which have listed biologic DMARDs on their NEMLs have disparate values for health expenditure per capita and SDI, the unifying factor appears to be the availability of established rheumatology services (adult and/or paediatric). As advocacy and demand are important in driving down the cost of these medicines, perhaps through pressure on manufacturers and funders, the role of rheumatologists to modernise care, has been demonstrated to be of particular importance in this setting.

However, a high similarity with the WHO EML does not necessarily translate to the direct availability of medicines for patients. Given the wide variation in GDP, health expenditure per capita, SDI and the appropriate prioritisation of communicable diseases in the WHO Africa region, improving care for rheumatic diseases in CYP remains complex. Many African countries have overburdened, heterogeneous healthcare systems which may include private health care and varying support from non-governmental organisations, where access to medicines may differ to that of patients dependent on state sponsored healthcare. Additionally, pharmaceutical services in institutions across Africa have required support from several globally funded programs to address operational gaps [43–46].

We therefore looked further to successful strategies that tackle high direct costs and the equitable distribution of medicines, that may be extrapolated to close ‘treatment gaps’ in this context.

In line with WHO Universal Health Coverage agendas (Sustainable Development Goal 3.8) and frameworks for the care of the chronically ill, the successful implementation of a universal access program for JIA in Chile as an example, resulted in timely diagnosis, higher rates of clinical remission and lower rates of complications [38]. This program was financed by a 1% increase in state value added tax; a strategy that may be difficult to implement in many regions of Africa, particularly after the devastating financial effects of the COVID-19 pandemic on many economies, as well as that of recent natural disasters, ongoing conflict and corruption [47, 48]. Nonetheless, the program highlights the positive effects of an enforced government mandated policy.

There are also several aspects of the successful multi-sectoral model for HIV/AIDS (WHO UNAID 3 by 5 initiative and others such as End TB and malaria) [49] which could be emulated in a globally driven strategy for rheumatic diseases in CYP. Tanzania and other East African countries implemented a ‘Non-Communicable Diseases Prevention and Control Programme’ in recent years, which reinforces multi-sectoral involvement and strong government commitment in effecting change [50, 51]. The system provides screening, prevention, diagnosis and treatment of hypertension and diabetes for people living with HIV, integrated with standard HIV healthcare services. Plans for extension to include those without HIV, is currently in progress. Supported by the WHO, and external funding (the United Nations program on HIV/AIDS (UNAIDS), the United States President’s Emergency Fund for Aids Relief (PEPFAR) and the Global Fund), these programs provide cost-effective care and medicines using existing infrastructure, with better outcomes as demonstrated in the INTE-Africa study [50–52].

In another positive step towards early recognition, diagnosis and treatment of rheumatic diseases in CYP, the Western Cape region of South Africa has included an extensive section on musculoskeletal disorders and inflammatory arthritis in the ‘Practical Approach and Care Kit for children’, a primary care manual complementing the WHO Integrated Management of Childhood Illnesses guideline and STGs [53].

While only 6 countries in sub-Saharan Africa have paediatric rheumatic diseases noted in STGs, this may be improved over time with lessons learned from these programs, and with adequate training and support, these models of primary care which includes the provision of appropriate medicines, could be successfully implemented for CYP with rheumatic diseases.

By prioritising rheumatic diseases and considering novel financing involving the Global Fund, managed entry agreements for new medicines, and the use of the Medicines Patent Pool as exemplified in these programs, medicines could be procured at a much lower cost and as part of a comprehensive package of care, affording patients and their families financial risk protection.

Pharmaceutical agreements may also encourage the expedited development of approved biosimilars (at a lower cost), paediatric medicine formulations and formulations which promote patient empowerment e.g. self-administered subcutaneous injections or oral medications, instead of intravenous administration. Oversight of patients requiring these medicines, including surveillance and treatment for TB and other infections, may pro-actively be achieved by adapting the monitoring systems already in place, as has been explored for other non-communicable diseases, and integrating telemedicine programs for paediatric rheumatology where in-person consultations are not feasible [54, 55]. These strategies may also be explored as potential avenues for the de-centralisation of care for CYP with rheumatic diseases once diagnosed, and the appropriate level of training has been achieved.

Maintaining up to date NEMLs would ensure the provision of a range of medicine for standard care. Countries which do not distinguish between the medicines listed for adults and children, or have medicines listed for rheumatic diseases but were updated before 2021, have the opportunity to advocate for ‘Medicines for musculoskeletal disorders’; and for the sub-section ‘Juvenile joint diseases’ to be aligned with the latest 2023 WHO model lists. For countries with an NEMLc, harmonising the medicines listed with the NEML and with the WHO model lists, is essential to ensure appropriate access to medicines for continuity of care throughout the life course.

## Conclusion

The safe and affordable provision of the range of medicines necessary to treat rheumatic diseases in CYP is a prerequisite for improving care and reducing disability. While countries in the WHO Africa region have worked to develop NEMLs, four countries (8.5%) have adequately updated their NEML since 2021 to reflect standard care for CYP with rheumatic diseases. Challenges to access and availability of medicines necessitate consolidation of efforts to align NEMLs with the WHO EML, and further include the development of healthcare policy to prioritise musculoskeletal disorders, the support of rheumatology services, and consideration of ways to integrate care with existing platforms for chronic diseases. Additionally, robust pharmaceutical management programs and surveillance are a key component for the safe, cost effective and affordable provision of the range of medicines necessary to treat rheumatic diseases in CYP.

### Limitations

This study is limited to NEMLs, a specifically defined mechanism for the procurement and supply of medicines for state health care services.

The authors recognise that medicines on the NEMLs may not be specifically listed for CYP with rheumatic diseases, but may still be accessible for use, and have made every effort to include all such medicines in this study, while acknowledging that some medicines may have been missed inadvertently.

The findings in this study cannot be extrapolated to the overall availability of medicines in a country, as medicines may be supplied via alternate pathways, e.g. private health care, or non-governmental organisations. Exploring these alternate avenues for the procurement of medicines were beyond the scope of this study and merits future analysis.

The findings in this study are further limited to the latest version of the NEMLs that were available online, which may not include the most recently released documents.

### Supplementary Information

Supplementary Material 1.

Supplementary Material 2.

## Data Availability

All data generated or analysed during this study are included in this published article (and its supplementary information files).

## Abbreviations

WHO: World Health Organisation
EML: Essential Medicines List
EMLc: Essential Medicines List for children
NEML: National Essential Medicines List
NEMLc: National Essential Medicines List for children
STG: Standard Treatment Guidelines
IQR: Interquartile range
GBD: Global Burden of Disease study
CYP: Children and young people
TF: Global Paediatric Musculoskeletal Task Force
LMIC: Low- and middle-income countries
DMARDs: Disease modifying anti-rheumatic drugs
TNFi: Tumour necrosis factor inhibitors
JIA: Juvenile Idiopathic Arthritis
