# Supplementary material for: A quantitative comparison between the essential medicines for rheumatic diseases in children and young people in Africa and the WHO model list
Source: Pediatr Rheumatol Online J. 2024 Jul 4;22:63. doi: 10.1186/s12969-024-00997-x (PMC11225199; doi:10.1186/s12969-024-00997-x)

# Supplementary Material

## Data extraction ( Supplementary Material Table 1)

The WHO EML and EMLc are organised alphabetically by indication. The *core* list presents the minimum medicine needed for priority conditions for a basic health-care system to function. The *complementary* list presents medicines for priority diseases for which specialised diagnosis and monitoring facilities and/or specialist medical care is needed.

To standardise comparisons and minimise the risk of missing data, the medicines for rheumatic diseases in children and young people were organised by section into a template for data extraction based on the 2021 WHO essential medicines model lists.

Biologic DMARDs were listed for juvenile idiopathic arthritis and were included in the section for ‘Juvenile Joint diseases’.

Table 1: Data extraction template

| WHO EMLc 2021 | Dosage form | Country  e.g Algeria | e.g Angola | e.g Benin |
| --- | --- | --- | --- | --- |
| *Section 29 Medicines for Diseases of Joints* | | | | |
| *Section 29.2 Disease-modifying anti-rheumatic drugs* | | | | |
| Methotrexate | Oral > Solid > tablet: 2.5 mg (as sodium salt) |  |  |  |
| Hydroxychloroquine | Oral > Solid: 200 mg (as sulphate) |  |  |  |
| Azathioprine | Oral > Solid > tablet: 50 mg (scored); 25 mg |  |  |  |
| *Section 29.3 Medicines for Juvenile Joint Diseases* | | | | |
| Acetylsalicylic acid | Oral > Solid: 100 to 500 mg Local > Rectal > Suppository: 50 to 150 mg |  |  |  |
| *Section 8.1 Immunomodulators for non-malignant disease as noted for rheumatic indication* | | | | |
| Adalimumab | Parenteral > General injections > SC: 40 mg per 0.8 mL; 40 mg per 0.4 mL; 10 mg per 0.2 mL; 20 mg per 0.4 mL |  |  |  |
| *Therapeutic Alternatives* | |  |  |  |
| Etanercept | Parenteral > General injections > SC: 25 mg per 0.5 mL; 50 mg per 1.0 mL |  |  |  |
| Infliximab | Parenteral > General injections > IV: 100 mg vial 10mg per 1.0 mL |  |  |  |
| No. of meds on NEMLc |  |  |  |  |
| % similarity with WHO EMLc |  |  |  |  |
| Medicines not on WHO EMLc |  |  |  |  |
| No. of meds NOT on WHO EML |  |  |  |  |
|  |  |  |  |  |
| WHO EML 2021 | Dosage Form |  |  |  |
| *Section 29 Medicines for diseases of joints* | | | | |
| *Section 29.2 Disease-modifying anti-rheumatic drugs* | | | | |
| Azathioprine | Oral > Solid > tablet: 50 mg (scored); 25 mg |  |  |  |
| ^c^Chloroquine | Oral > Solid: 100 mg tablet (as phosphate or sulfate); 150 mg tablet (as phosphate or sulfate) |  |  |  |
| Hydroxychloroquine | Oral > Solid: 200 mg (as sulfate) |  |  |  |
| Methotrexate | Oral > Solid > tablet: 2.5 mg (as sodium salt) |  |  |  |
| Penicillamine | Oral > Solid: 250 mg |  |  |  |
| Sulfasalazine | Oral > Solid: 500 mg |  |  |  |
| *Section 8.1 Immunomodulators for non-malignant diseases* | | | | |
| Adalimumab | Parenteral > General injections > SC: 40 mg per 0.8 mL; 40 mg per 0.4 mL; 10 mg per 0.2 mL; 20 mg per 0.4 mL |  |  |  |
| *Therapeutic Alternatives* | |  |  |  |
| Certolizumab pegol | Parenteral > General injections > SC: 200 mg per 1.0 mL |  |  |  |
| Etanercept | Parenteral > General injections > SC: 25 mg per 0.5 mL; 50 mg per 1.0 mL |  |  |  |
| Golimumab | Parenteral > General injections > SC: 50 mg per 0.5 mL; 45mg/0.5ml> IV: 50mg/4ml |  |  |  |
| Infliximab | Parenteral > General injections > IV: 100 mg vial 10mg per 1.0 mL |  |  |  |
| No. of meds on NEML |  |  |  |  |
| % similarity |  |  |  |  |
| Medicines NOT on WHO EML |  |  |  |  |
| No. of meds NOT on WHO EML |  |  |  |  |
| *WHO EML Section 29.3 Juvenile Joint Diseases* | |  |  |  |
| *Section 29 Medicines for diseases of joints* | |  |  |  |
| *Section 29.2 Disease-modifying anti-rheumatic drugs* | |  |  |  |
| Methotrexate | Oral > Solid > tablet: 2.5 mg (as sodium salt) |  |  |  |
| *Section 8.1 Immunomodulators for non-malignant diseases* | |  |  |  |
| Adalimumab | Parenteral > General injections > SC: 40 mg per 0.8 mL; 40 mg per 0.4 mL; 10 mg per 0.2 mL; 20 mg per 0.4 mL |  |  |  |
| *Therapeutic Alternatives* | |  |  |  |
| Certolizumab pegol | Parenteral > General injections > SC: 200 mg per 1.0 mL |  |  |  |
| Etanercept | Parenteral > General injections > SC: 25 mg per 0.5 mL; 50 mg per 1.0 mL |  |  |  |
| Golimumab | Parenteral > General injections > SC: 50 mg per 0.5 mL; 45mg/0.5ml> IV: 50mg/4ml |  |  |  |
| Infliximab | Parenteral > General injections > IV: 100 mg vial 10mg per 1.0 mL |  |  |  |
| Acetyl salicylic acid | Oral > Solid: 100 to 500 mg Local > Rectal > Suppository: 50 to 150 mg |  |  |  |
| No. of meds on NEML section Juvenile joint diseases | |  |  |  |
| % similarity |  |  |  |  |
| Medicines not on WHO EML section Juvenile joint diseases | |  |  |  |
| No. of meds not on WHO EML section Juvenile joint diseases | |  |  |  |

^c^ Medicine on core WHO EML- All other medicines on complementary list

| Biologic DMARDs | Acetyl salicylic Acid | Conventional Synthetic DMARDs |
| --- | --- | --- |

## Fig. 1: Summary of the number of African countries with the essential medicines for rheumatic diseases in children and young people as on the WHO EML

*Conventional synthetic DMARDs were available more commonly on NEML’s as compared to tumour necrosis factor inhibitors*

## Interactive map of the Essential medicines for rheumatic diseases in children and young people

Available at [*https://batchgeo.com/map/53d21397fc51bcd1f309d64e75c9e81f*](https://batchgeo.com/map/53d21397fc51bcd1f309d64e75c9e81f)

Figure 1: Screenshot of the example for Algeria


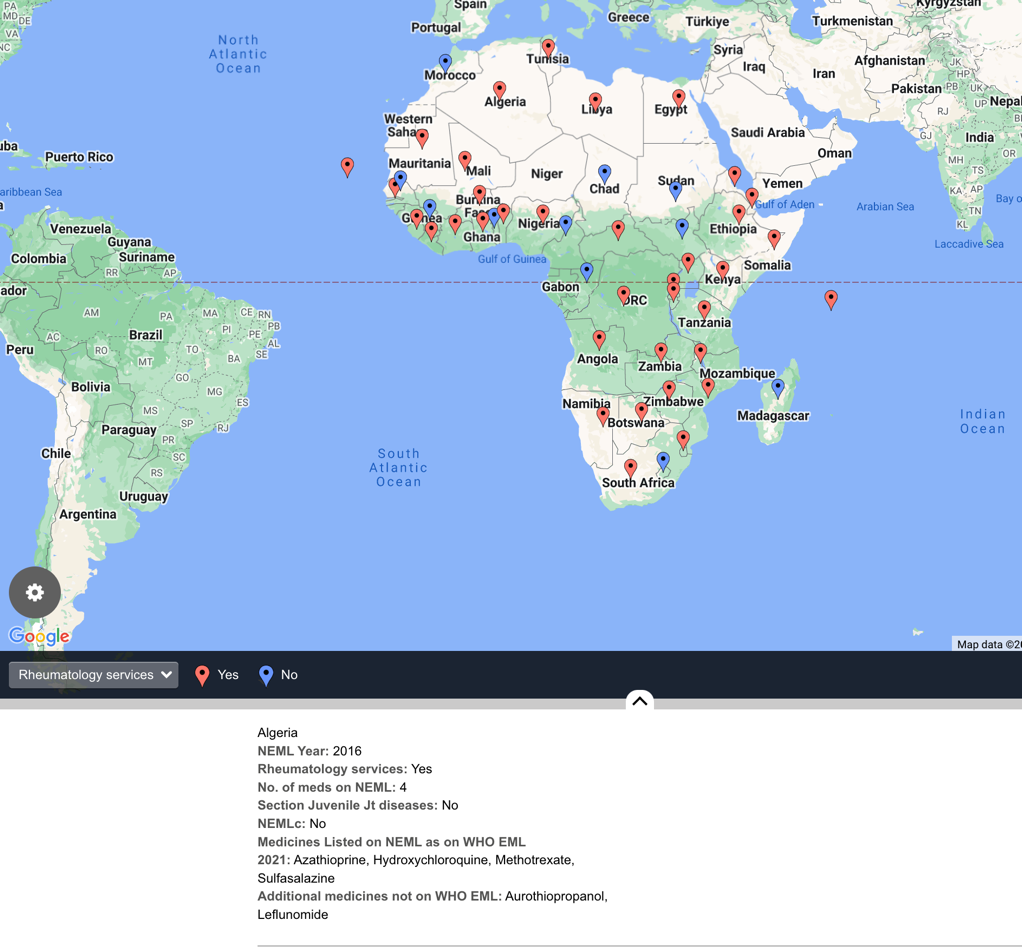

Supplement: Supplementary file 1 — Supplementary Material 1. [file 12969_2024_997_MOESM1_ESM.docx]
